# Communication and marketing as tools to cultivate the public's health: a proposed "people and places" framework

**DOI:** 10.1186/1471-2458-7-88

**Published:** 2007-05-22

**Authors:** Edward W Maibach, Lorien C Abroms, Mark Marosits

**Affiliations:** 1 Department of Prevention and Community Health, George Washington University School of Public Health & Health Services, 2175 K Street, Suite 700, Washington, DC, 20037, USA; 2 Worldways Social Marketing, 6030 Greenwood Plaza Blvd., Suite 110, Greenwood Village, CO, 80111, USA

## Abstract

**Background:**

Communication and marketing are rapidly becoming recognized as core functions, or core competencies, in the field of public health. Although these disciplines have fostered considerable academic inquiry, a coherent sense of precisely how these disciplines can inform the practice of public health has been slower to emerge.

**Discussion:**

In this article we propose a framework – based on contemporary ecological models of health – to explain how communication and marketing can be used to advance public health objectives. The framework identifies the attributes of people (as individuals, as social networks, and as communities or populations) and places that influence health behaviors and health. Communication, i.e., the provision of information, can be used in a variety of ways to foster beneficial change among both people (e.g., activating social support for smoking cessation among peers) and places (e.g., convincing city officials to ban smoking in public venues). Similarly, marketing, i.e., the development, distribution and promotion of products and services, can be used to foster beneficial change among both people (e.g., by making nicotine replacement therapy more accessible and affordable) and places (e.g., by providing city officials with model anti-tobacco legislation that can be adapted for use in their jurisdiction).

**Summary:**

Public health agencies that use their communication and marketing resources effectively to support people in making healthful decisions and to foster health-promoting environments have considerable opportunity to advance the public's health, even within the constraints of their current resource base.

## Background

Communication is rapidly coming to be recognized as a core function, or core competency, in the field of public health. Several developments over the past few years illustrate this fact. In 2003, the Institute of Medicine identified communication as a core public health competency and called for efforts to enhance the communication skills of the public health workforce.[1] Over the past five years the National Cancer Institute – the largest biomedical research funding agency in the U.S. – has significantly increased the size of its health communication research portfolio after identifying health communication as vital to future progress in cancer control.[2]

In 2005, the Directors-General of National Public Health Institutes (NPHIs) – technical assistance units established within national health ministries – identified health communication as a core function of NPHIs,[3] and the Pan American Health Organization committed to "better utilize or increase, if needed, the numbers of ... communication experts" working in its member organizations.[4] Between 2004 and 2006, several U.S. schools of public health launched Masters in Public Health (MPH) degree programs in public health communication[5-7] – which added significant new training capacity on top of the one extant program[8] – and the U.S. Association of Schools of Public Health published a draft set of communication competencies that are proposed to be required of every Masters in Public Health (MPH) graduate from accredited U.S schools of public health.[9]

Although marketing has not been formally recognized as a core public health function or competency – possibly because negative associations toward the concept by some in public health as a result of its roots in the business sector – many leading public health organizations are seeing its relevance to public health purposes and building their capacity in this discipline. Health Canada first established it Social Marketing Unit in 1981 and continues to expand its social marketing expertise.[10] The U.S. Centers for Disease Control and Prevention established the National Center for Health Marketing in 2004,[11] and a number of U.S. states – Arizona, California, Ohio and North Carolina, at a minimum – have recently established social marketing units. The National Health Service in the UK is currently considering a proposal to integrate social marketing as a core strategy in managing the health of the British population,[12] and public health organizations in the pacific region are working to enhance their marketing capacity.[13]

Health communication and social marketing have been vibrant areas of academic research and professional practice for several decades,[14,15] with both areas of inquiry yielding dedicated journals,[16,17] numerous books,[18-21] and myriad peer-reviewed manuscripts published in public health journals.[22-24] What has been slower to emerge, however, is a coherent sense of precisely how these disciplines can inform the practice of public health.

In this article we propose a framework through which to understand how to effectively harness the tools of communication and marketing in the practice of public health. We do so by first proposing a simple framework for public health action, and then by demonstrating the relevance of communication and marketing within the proposed framework.

## Discussion

### The context: ecological models of health

The Ottawa Charter[25] was a turning point in public health in that it prefaced a sea change in how public health professionals think about promoting health.[26] Its legacy – and that of leading epidemiology and population health theorists in the early 1990s[27-30] – can be seen clearly in contemporary ecological models of health.[31-34]

The concept of ecology "pertains broadly to the interrelations between organisms and their environments."[35] We interpret ecological models of health as positing, in essence, that the health of populations is influenced by: (a) the attributes of the people in the population; (b) the attributes of the environments – or places – in which members of the population live, work, go to school, shop and so forth; and (c) important interactions between the attributes of people and places. These attributes and their interactions typically influence health through their impact on health behavior and through direct effects on physical functioning and well-being.[36-42]

Health, and its behavioral, social, and environmental determinants, is nothing if not complex. A recent effort by Sallis and colleagues to create an ecological model of "active living" – i.e. physical activity – provides an excellent example of an attempt to capture this complexity.[43] Their model identifies seven broad categories of individual and environmental variables (intrapersonal, social cultural environment, natural environment, information environment, perceived environment, policy environment, and access to and characteristics of behavior settings) that influence active living behavior in each of four domains of active living (transportation, recreation, household activities, occupational activities).

**Figure 1:**
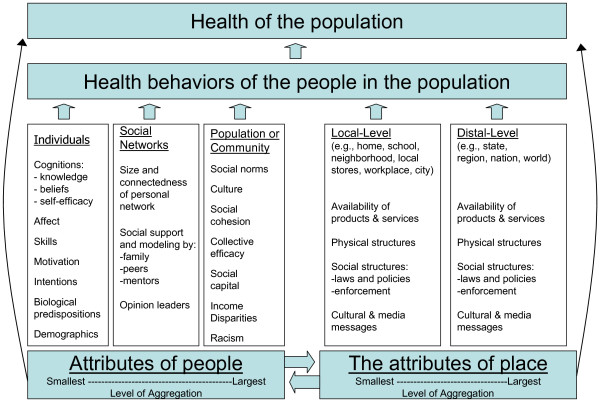
A people and places framework for public health influence.

In the spirit of Einstein's famous dictum – "Everything should be made as simple as possible, but not simpler"- we propose a streamlined ecological model of public health action that we call the People & Places Framework. By design, our framework principally calls attention to the attributes of people, and the attributes of places, that are known to influence the health behavior, and health, of populations. These two orthogonal factors – people and places – each operate across two or three relevant levels of analysis. Our framework describes the relevant attributes of people as operating in individual, social network, and community or population levels of analysis, and the relevant attributes of place as operating in local and distal levels of analysis). The framework is illustrated in Figure 1 and is described below. To simplify the visual presentation, we laid out the people and place factors side-by-side rather than orthogonally.

Throughout the remainder of the paper, we refer to these levels of analysis as "fields of influence" because our objective here is to describe a framework for public health action rather than a theory or theoretical framework for research purposes.

#### People-based fields of influence

Renaissance era author John Donne's famous quote – "no man is an island, entire of itself; every man is a piece of the continent" – was prescient in its foreshadowing of contemporary theories of social science. These theories make clear that people exist within various levels of aggregation.[44] Building on earlier work, we propose that three levels of social aggregation, specifically the individual-level, social network-level, and group-, community- or population-level, offer a useful and parsimonious means of categorizing people-based fields of influence.[45]

Individual-level factors that influence health and health behavior have been the subject of intense research activity for many decades, particularly in various fields of psychology, communication research, and epidemiology. There has been – and will continue to be – lively debate regarding which individual-level factors are most relevant with regard to influencing health behavior and health.[46] Our point here, however, is not to take a position on that debate, but rather simply to affirm the importance of individual-level factors as an important field of influence on health behavior and health. For purposes of illustration, the literature points to the following as relevant individual-level attributes: cognitions (e.g., self-efficacy and outcome expectancies),[47] affect (e.g., depression),[48] skills (e.g. contraceptive skills),[49] motivation,[50,51] intentions,[52] biological predispositions (e.g., sensation seeking),[53] and demographic factors (e.g., marital status, education, income, employment status).[54,55] Most of these attributes are amenable to change through external intervention.

Much is also known about the relevant attributes of social networks with regard to health behavior and health.[56] Insights into the influence of social networks come from fields as diverse as sociology, communication, psychology, and business and organizational studies. Again, our purpose here is not to promulgate a specific theory or definitive list of attributes, but rather to affirm the importance of social networks as a field of influence on health behavior and health. The literature suggests that the relevant attributes of social networks, at a minimum, include: size and connectedness of a person's social network,[57] diversity of ties in the social network,[58] the degree to which the various relations in a social network (e.g., parents, friends, teachers and mentors) provide social support[59-62]and positive modelling,[63] and the presence of positive health opinion leaders in the social network.[64-67]

Even though Durkheim's seminal work over a century ago illustrated the importance of population attributes on health (in particular on suicide rates), the relevant attributes of groups, communities and populations with regard to health behaviors and health are perhaps the least well understood.[68] Culture and social norms are important, well documented attributes of communities and populations, although it can be argued that these are attributes operate at the individual and social network levels as well.[69] A rapidly emerging literature suggests that other important attributes of groups, communities and populations include social capital,[70] social cohesion,[71] and collective efficacy,[72-74] although additional work is needed to explicate and operationally define each of these attributes. Additionally, a large and rapidly growing body of literature is elucidating how socio-economic disparities – particularly the income gap between the most well-off and least well-off members of a community – and racism exert an important negative influence on health.[75-78]

#### Place-based fields of influence

Needless to say, people and places are inextricably linked. Tom Farley and Deborah Cohen open their book *Prescription for a Healthy Nation *with a trenchant quote from Winston Churchill to illustrate this point: "We shape our buildings, and thereafter they shape us."[79] The influence of place – including our homes, schools, worksites, roads, food markets and restaurants, neighbourhood, cities, and so on – manifests itself on our health behavior, and health, in myriad complex ways. Cohen, Farley and Scribner developed a simple, elegant way to categorize these place-based influences into four factors: [80]

• *The availability of products and services*. Increased availability of health enhancing products and services (e.g., primary health care, fresh produce) tends to promote population health, while increased availability of health detracting products and services (e.g., liquor stores) has a tendency to undermine population health.

• *The physical structures in our environment*. Structures that as a natural by-product of their design encourage healthful actions (e.g., sidewalks, walking paths, easily accessible stairwells) or discourage unhealthful actions (e.g., reduced serving sizes) or outcomes (e.g., automobile airbags) tend to promote population health. Conversely, structures that as a natural by-product of their design promote unhealthful actions (e.g., super-sized meals, televisions) or enable actions that lead to morbidity or mortality (e.g., poor roadway design) tend to undermine population health.

• *The social structures (i.e., laws and policies) in our communities, and the extent to which they are enforced*. Laws and policies that require (e.g., seatbelt and child safety restraint laws) or encourage (e.g., enhanced access to fruits and vegetables in schools) healthful action, and those that discourage unhealthful actions (e.g., high tobacco taxes) tend to promote population health. Conversely, laws and policies that intentionally or inadvertently enable unhealthful behavior (e.g., permissive alcohol sales regulations) tend to undermine population health.

• *The media and cultural messages in our environment*. Media and cultural messages which model and recommend healthful practices (e.g. advertising which promotes fruit and vegetable intake) tend to promote population health, while media and cultural messages which model or promote behaviors ill-conducive to health (e.g. advertising which promotes intake of foods high in fats and sugars) tend to undermine population health.

These place-based factors operate both locally (e.g., within our own home, and in our city), and more distally (e.g., from actions taken in our state capital, in our nation's capital, and by multi-national corporations and multi-national governmental organizations).[81,82] Decisions made (or not made) in local places exert influence in a variety of pervasive ways over the behavior and health of people in that one location.[83] Conversely, the decisions made in distal places – e.g., Hollywood, Wall Street, Washington, DC – often have the potential to influence people's behavior and health over large geographic regions. Therefore, our proposed framework differentiates local and distal environments as distinct fields of influence.

The remainder of this paper focuses on describing the relevance of communication and marketing to public health practice through the lens of the People & Places Framework.

### Definitions of communication and marketing

The distinction between communication and marketing is poorly understood throughout the field of public health.[84,85] They are often seen as interchangeable.[86] We believe, however, that the concepts are distinct and that the distinctions are meaningful for public health. Each method offers a different and complementary approach through which to advance public health objectives.

Finnegan and Viswanth[87] – based on earlier writing by Gerbner[88] – provide a useful and concise definition of the act of communication as "the production and exchange of information and meaning by use of signs and symbols." Healthy People 2010 – a publication that presents the current U.S. federal health objectives – defined health communication as "the art and technique of informing, influencing and motivating individual, institutional and public audiences about important health issues."[89] This definition is laudable for its inclusion of the full range of audiences implied by an ecological framework. Borrowing from the strengths of each definition, we define health communication as "the production and exchange of information to inform, influence or motivate individual, institutional and public audiences about health issues."

The American Marketing Association defines marketing as "an organizational function and a set of processes for creating, communicating, and delivering value to customers and for managing customer relationships in ways that benefit the organization and its stakeholders."[90] Inherent in this definition is the notion of the marketing exchange. The organization delivers value to the customer, usually in the form of products or services, in exchange for the customer's resources, usually in the form of money, effort and/or time, and which go on to benefit the organization. This definition makes clear that marketing involves the process of communication, but only as integrated function focused on creating and delivering value to customers through products and services.

Marketing and communication do overlap, both in concept and in how they are applied in public health. Marketing communication, or promotion, involves the use of communication to support the marketing process. Specifically, marketing communication is used to inform prospective customers, and business partners, about the availability, benefits, and costs associated with the organization's products and services, and to manage relationships with those key stakeholders. Moreover, the practice of public health communication has been greatly influenced by marketing methods, especially the use of marketing research and adoption of a consumer-orientation. Despite these areas of overlap, we believe that marketing and communication are sufficiently distinct – with distinctions that are directly relevant to effective public health practice – as to necessitate that one activity not be considered a sub-set of the other. The definitions above were provided with the specific intent of clarifying confusion in the literature where communication has often been mistaken for marketing, and vice versa.

### The relevance of communication and marketing in the people & places framework

**Figure 2:**
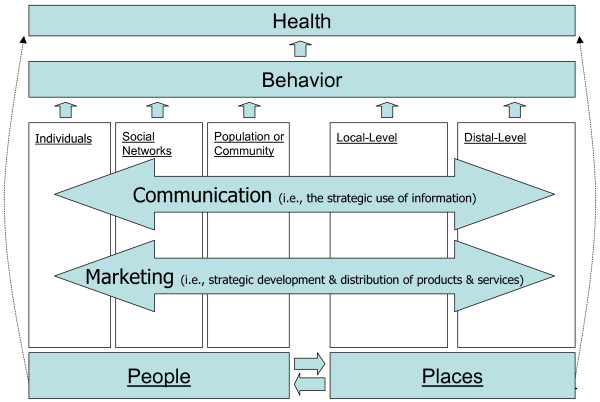
The Relevance of communication and marketing in the People & Places Framework.

**Figure 3:**
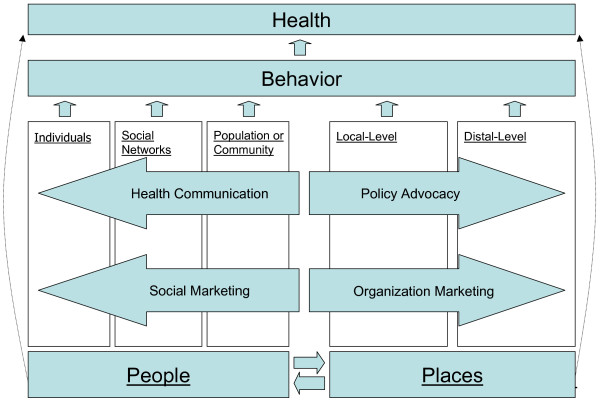
The specific roles of communication and marketing in the People & Places Framework.

One metric by which to gauge the relevance of communication and marketing to public health practice is the extent to which they are capable of creating – or contributing to – beneficial changes in each of the five fields of influence. Figure 2 illustrates our contention that communication and marketing each have potential to contribute to beneficial changes in all five fields of influence, and Figure 3 identifies the specific uses, or roles, of communication and marketing as they have been explored in public health to date. We explore each of these specific uses of communication and marketing below.

### Using communication to create change in people-based fields of influence

#### Individuals

Often referred to as "health communication," the use of communication methods to provide individuals with important health information has been a part of public health practice for decades, if not centuries. In the early 1700s, for example, Cotton Mather mounted a communication campaign in Boston to promote smallpox inoculation.[91] Informing people about immunizations remains an important public health communication priority today.[92-94]

A rich set of theories – mostly drawn from the fields of social and cognitive psychology – have been used successfully in developing health messages for individuals.[95,96] These theories include Social Cognitive Theory,[97] Elaboration Likelihood Model,[98] Stages of Change Theory,[99] and Theory of Reasoned Action,[100] and Persuasion Theory.[101] More recently, Fischhoff and colleague's "mental models" approach,[102] and other newly developing theories based in the emerging findings of neuroscience have provided new insights into effective health communication.[103]

The type and availability of communication vehicles that can be used to convey public health information to individuals has grown dramatically over time.[104] Some of these communication vehicles – e.g., brochures, small group counselling sessions, interactive DVDs, email and text messages – are well-suited to providing information to individuals on a one-to-one, or a one-to-few, basis. These communication vehicles can be effectively tailored to respond to individual attributes of the person receiving the information.[105] Other communication vehicles – e.g., TV, radio, newspapers, movies, websites – are well-suited to providing information on a one-to-many, or mass, basis. It is worth noting, however, that when we use communication vehicles on a "one-to-many" basis, we are typically attempting to influence individual-level attributes of people (e.g., self-efficacy) *en mass*, rather than attempting to influence attributes of the population per se (e.g., collective efficacy). The latter use of communication, as explained below, is a relatively unexplored opportunity in public health.

The dominant communication question of interest among health researchers and practitioners focused on the individual field of influence is: *Can we use messages to influence people in beneficial ways? *The answer to this question appears to be a qualified "yes." Case study evidence,[106] meta-analysis,[107] and systematic literature reviews[108-110] have each recently concluded that public health communication initiatives are, on the whole, effective in changing people's behavior, but usually only modestly so. To succeed, public health communication initiatives must be heard and remembered against the din of other competing messages in the media.[111] Many health communication campaigns have failed, however, because they did not achieve adequate "reach and frequency" and were not able to reliably expose members of the target audience to campaign messages.[112]

That the average public health communication campaign is only modestly effective, however, stands to reason when viewed in the context of an ecological model of health. Public health efforts to influence a single field of influence – in this case, individuals – will, on average, have only limited success because, in most cases, the other fields of influence also play significant roles in shaping the relevant behaviors and health outcomes. There are clear exceptions to this rule, however. Sudden Infant Death Syndrome (SIDS) campaigns in various nations around the world stand as an example of where communication efforts to create change in the individual field of influence (in this case by encouraging parents to place their babies to sleep on their backs) have been sufficient to create dramatic behavior change and improvements in health outcomes.[113] Presumably, communication to individuals in these cases was sufficient to create large-scale and sustained behavior change because parents are highly motivated to protect the health of their newborns, few social network, group, or environmental barriers stood in the way of their behavior change, and the behavior being recommended is quite easy to perform. The "truth" youth anti-smoking campaign is another apt exception to the rule. [114]

#### Social networks

The potential influence of other people – rather than the potential influence of messages – has been the dominant question of interest among researchers and practitioners focused on the social network field of influence. Diffusion of Innovation theory[115,116] (as developed over the course of five decades by Everett Rogers and recently launched into popular culture by Malcom Gladwell's book The Tipping Point[117]) has been particularly influential and helpful in highlighting for public health audiences the importance of social networks, although this research has tended to be explanatory rather than interventional in nature. Related areas of research have focused on activating existing relationships within social networks or developing new social networks in ways that enhance the provision of useful health information, positive sources of influence, and social support. The essential question is: *Can we influence social networks so that they promote health?*

Activating people within existing social networks to serve as agents of behavior change has proven to be a productive approach for cultivating health enhancement. Popular peers,[118,119] spouses,[120] parents of adolescents,[121] lay health workers and health care providers[122] have all been shown to have important behavioral influence on others.

Relatively less-studied is the question: *Can we use communication to activate people to serve as agents of positive health influence within their social networks? *The largest US health communication campaign to date, the Youth Anti Drug Media Campaign, is currently attempting to activate parents to take actions that are known to reduce the likelihood that their children will use drugs.[123] The initiative thus far has had only limited success in eliciting the recommended parenting behaviors.[124] An earlier effort, conducted largely through outreach to producers, directors and writers in Hollywood successfully promoted the designated driver concept as a way to reduce driving under the influence of alcohol.[125]

Interpersonal influence between peers, family members, and other members of social networks (i.e., "word of mouth" influence) – and interventions that attempt to harness this influence – has historically occurred primarily through face-to-face interaction. The introduction of the telephone in the 20th century added a new vehicle through which interpersonal influence could be expressed. Now, the internet is creating significant new possibilities for word of mouth influence.[126] The internet allows people to expand and strengthen their social networks.[127] Most recently, the growth of "social media" on the internet – places where social networks form in a manner not bounded by geographical constraints – has added a new and rapidly growing dimension to this field of influence. For example, MySpace – which lets people meet and interact with others who share similar interests and share content they create themselves such as blogs, photos, and videos – is currently the most popular destination on the Web.[128] The implications for harnessing this growing social influence process to advance the public's health are only now beginning to be considered.[129]

#### Groups, communities and populations

Understanding the influence of group, community and population attributes on health is a rapidly blossoming area of public health inquiry. Research focused on identifying viable means to influence these relevant attributes of communities and populations, however, is still in early stages of development. Important exceptions include the long-standing traditions of community organizing[130] and coalition building,[131] and the more recently established community-based participatory research model.[132,133]

The question of interest here is: *Can we use communication to cultivate the attributes of community or population that promote health? *Wallack has begun to articulate an answer to this question by identifying civic journalism and photovoice as promising approaches for using the mass media to build social capital in communities.[134] Civic journalism is the use of journalism to engage the community in the process of civic life.[135] Its methods – involving a variety of types of data gathering from, and information presentation to, members of the community – are intended to increase community debate and public participation in problem solving.[136] The preliminary evidence indicates that these methods offer an effective means for engaging community members in addressing important problems in their community.[137]

An interesting example of civic journalism was implemented during the most recent US presidential primary campaign. Rock the Vote, a national youth vote organization, partnered with CNN to sponsor a nationally-televised debate where Democratic presidential candidates responded to questions posed directly by young citizens. Young viewers of this event experienced greater identification with the candidates and enjoyed a heightened level of political efficacy as compared to young viewers of a traditional journalist-led debate format.[138] In other words, the positive impact of this exercise in political engagement was heightened simply by allowing members of a politically disenfranchised group (rather than a paid professional) to pose the questions to candidates.

Photovoice is a process that engages members of a community – typically members of a marginalized community – in using photography to document a public health problem that disproportionately affects them, from their own perspective.[139,140] This method seeks to encourage and enable members of the community to act on their own behalf. To the extent that it succeeds in doing so, this method can have a beneficial impact on important attributes of the community field of influence. A second and equally important objective of the method – engaging policy makers and other community leaders in the issue of concern so that they will effect the changes as recommended by members of the afflicted community – is perhaps better thought of as a place-based change strategy (see the "Using Communication to Shape the Place-Based Fields" section below).

### Using marketing to create change in the people-based fields of influence

#### Individuals

Typically called "social marketing," the use of marketing to elicit health behavior change from individuals has been an active area of practice and research for the past several decades.[141,142] Much of what is called "social marketing" by practitioners and academics is not marketing, however, because neither products nor services are developed, distributed, or promoted. Rather, most of what is referred to as social marketing in public health involves exclusively the provision of information, and is therefore more correctly characterized as communication.[143]

The question of interest is: *Can we develop and deliver products or services that will elicit the behavior we seek from members of our target audience? *An excellent example, cited in a recent review by Grier and Bryant, illustrates how a social marketing program can reduce the incidence of driving under the influence of alcohol.[144] To address the problem of high rates of alcohol-impaired driving among young men in rural areas, the Wisconsin Department of Transportation conducted qualitative research with members of this group. In focus group interviews, the young men indicated they would not be dissuaded from drinking with their friends in bars after work, but they expressed concern about the risks associated with driving themselves home at the end of the evening. In response, the Department of Transportation developed a fee-based taxi service – The Road Crew – to safely transport people who have been drinking (or plan to drink) so that they do not drive themselves. In its first year, the marketing program proved popular with members of its target audience (over 17,000 rides were provided), and it earned widespread support from the communities where the program is offered, in part, because it reduced alcohol-related crashes by 17%.[145] Other examples of successful social marketing initiatives include the distribution and sale of condoms[146] and other contraceptives,[147] oral rehydration therapy,[148] bed nets to families in areas afflicted by high levels of malarial infestations,[149] and the distribution of point-of-use safe water products to prevent diarrhoeal disease in areas without adequate water sanitation facilities.[150]

#### Social networks

A less explored use of social marketing involves developing and delivering products or services that target key members of social networks whose actions can benefit other members of their social network. The question of interest is: *Can we use marketing to enhance or influence social networks so that they promote health? *Kelly and colleagues' Popular Opinion Leader (POL) HIV prevention intervention provides an interesting example.[151] POL interventions attempt to influence a given geographically-bounded social network or community. The methodology involves recruiting approximately 15% of the members of a social network over time – specifically, those people who are most popular and trusted by others in the social network – into HIV prevention and advocacy training that is administered through multiple small-group sessions. When successfully implemented, the program results in significant community-wide rates of HIV risk reduction by virtue of the social influence brought to bear by the popular opinion leaders in the community.[152,153]

#### Groups, communities and populations

Public health professionals have only recently begun to consider the potential of social marketing to influence important attributes of groups, communities and populations. In theory, products and services that make it easier for citizens and community organizations to successfully come together around a common purpose and engage in community change efforts should promote both collective efficacy and social capital.[154-156] The question, therefore, is: *Can we develop and deliver products or services that promote the community-level attributes that enhance health?*

The Gatehouse Project in Australia provides an intriguing example.[157] To promote greater social inclusion and sense of school connectedness among the entire student population of 12 secondary schools, the project's personnel provided school officials with training and feedback, and a student curriculum, aimed at improving the school's social climate. These activities had a significant positive impact. Two years after the completion of the intervention, community-wide rates of substance use, anti-social behavior, and sexual intercourse were 25 percent lower in intervention schools as compared to control schools.

### Using communication to shape the place-based fields of influence

The past several decades have been a time of considerable foment with regard to the uses of communication to positively influence environments. Policy advocacy – often referred to as "media advocacy" – has emerged as an important communication-based public health intervention modality. The key question here is: *Can we use communication to promote beneficial changes in the places that influence peoples' health? *We address this question as it pertains to both local- and distal-level places in a single discussion below, because the approaches are similar regardless of level of analysis.

Media advocacy has been defined as "the strategic use of mass media in combination with community organizing to advance healthy public policies."[158] Media advocacy involves framing public health issues, and creating news, so that members of a community will take notice, and take action, to force policy makers to revise the policies that are giving rise to the problem.

There is growing evidence supporting the effectiveness of this approach, especially at the local level.[159,160] The largest systematic effort to test policy advocacy methods to date – ASSIST (American Stop Smoking Intervention Study), a policy change-oriented tobacco control intervention conducted in 17 US states – demonstrated increased coverage of tobacco control issues in ASSIST states, including greater coverage of tobacco policy issues, although the increases in media coverage were smaller than was expected.[161]

Policy advocacy methods can also be used to target private sector policy makers who make myriad important decisions that affect the health of their stakeholders and the public at large (e.g., the CEO of Wal-Mart and other major corporations). For example, flight attendants and their union played an important role in getting smoking banned from airliners, the first ban on smoking in the workplace in the US.[162] Northwest Airline responded with a decision to prohibit smoking on all North American flights, several years in advance of being required to do so by law.[163]

Currently, public health advocacy efforts targeting the soft drink industry appear to be having positive influence. In recent years both Coca-Cola and Pepsi-Cola adopted policies to prevent their sodas from being sold in elementary schools in the US. More recently, the American Beverage Association (ABA), whose members include virtually all soft drink manufacturers, collaborated with American Heart Association and the William J. Clinton Foundation to create a voluntary set of policies that will, if embraced by ABA members, further limit soda sales in schools.[164] ABA's target is to have the policies honoured by their members in 100 percent of US schools by the beginning of the 2009–2010 school year.

### Using marketing to shape the place-based fields of influence

Organization marketing (which in the literature is frequently referred to as business marketing or business-to-business marketing) – the process of marketing to potential customers in businesses, government agencies, and non-profit organizations – is an important use of marketing that is distinct both from consumer marketing and from traditional forms of social marketing. Andreasen[165] and Maibach and colleagues[166,167] have recently proposed the need for public health professionals to embrace organization marketing for its potential to positively influence environments and create "upstream" (i.e., place-based) changes conducive to the public's health. The key question is: *Can we use marketing to promote beneficial changes in the places that influence peoples' health? *A number of recent examples illustrate the potential.

The Popular Opinion Leader (POL) intervention for HIV prevention, as discussed above, has proven to be a highly effective in reducing population risk for HIV infection.[168] To encourage its broader adoption and use, Kelly and colleagues developed a web-based means of marketing the POL intervention to HIV prevention organizations in communities around the world. To evaluate this marketing approach, they specifically targeted HIV prevention organizations in 78 nations.[169] The marketing program was highly successful in that approximately 70 percent of the organizations that received the marketing offer adopted the POL intervention in their communities, or trained other agencies to use it.

To encourage other organizations (e.g., county health departments, school districts) to adopt proven disease-prevention programs, rather than use unproven programs, several US federal and non-profit health agencies created Cancer Control PLANET. PLANET is an online marketplace designed to facilitate the selection of evidence-based cancer prevention programs that are available for adoption by other organizations.[170] To enhance PLANET's value to potential customers, these agencies are currently taking active steps to expand their online library of proven programs. The US Centers for Disease Control and Prevention's DEBI (Diffusing Evidence-Based Interventions) Project is a conceptually similar effort to market evidence-based HIV prevention programs.[171]

**Figure 4:**
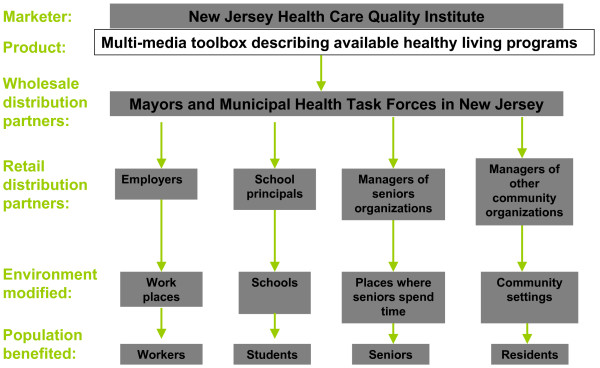
Distribution channels for the New Jersey Mayors Wellness Campaign.

Another excellent example is a newly-launched organizational marketing initiative by the New Jersey Health Care Quality Institute which is attempting to market evidence-based approaches to health enhancement – programs and modifications to the built environment – to New Jersey's mayors and municipal health task forces. The objective is to enable mayors and municipal task forces to, in turn, market the programs to employers, school officials, senior care managers, and community-based organizations in their cities. The logic model underlying this organization marketing strategy is illustrated in Figure 4.

### The importance of cultivating change in multiple fields of influence

Inherent in the logic of ecological models of health is the premise that, to have the largest impact on behavior and health, public health professionals should seek to create change, as feasible, among multiple levels of influence.[172] Confirmation of this premise is nicely illustrated in the context of food micronutrient fortification programs.

Food micronutrient fortification strategies have long been the preferred strategy to ensure that people's diets include an adequate micronutrient levels precisely because of the assumption that "fortification requires minimal consumer involvement and little to no change in dietary habits."[173] A recent review of fortification programs, however, concluded that "the primary factor leading to long-term sustainability of food fortification is consumer awareness of the nutrient deficiency and consumer demand for and perceived benefits of the fortified food." This illustrates that the domains of influence reinforce one another, and that cultivating change in both people- and place-based fields of influence enhances the odds of achieving population-based health gains.

Of course, public health resources are inherently scarce and they must be stewarded wisely.[174] Organizations in the public health sector cannot afford to, nor would they be wise to, invest indiscriminately in attempting to create change in all fields of influence for every public health problem. We believe that public health organizations should develop strategic plans that consider and balance the following four factors in determining how to focus their efforts. These factors are: (1) the organization's current and potential resources; (2) the relative importance of each field of influence in creating or sustaining the problem being addressed; (3) the likely impact (and other potential benefits) associated with various programmatic options that the organization is capable of implementing; and (4) the likely costs of implementing the various programmatic options under consideration. This approach is grounded in well-established methods for health promotion program planning and management,[175] as well as in more recent thinking about how best to focus programmatic efforts against public health challenges with dimensions that are both people- and place-based.[176] Describing various methods for considering these factors is beyond the scope of this paper.

### Communication and marketing are assets in dissemination of evidence-based public health programs

Over the past few decades, there has been a growing recognition of the importance of the dissemination of evidence-based "best practices" in public health programs, practices, and policies.[177] Thus far, however, the notion of evidence-based public health has been more promise than practice. Communication and marketing methods offer great potential in helping to bridge the divide between the promise and the practice of evidence-based public health.[178]

Conceptually, the challenges are fairly straight-forward: encouraging practitioners and policy makers to factor the evidence-base into their decision-making; and making it easy for them to effectively do so.[179] To fully harness the value of the evidence-based public health paradigm, however, we must successfully address these dissemination challenges with many different types of practitioners and decision-makers. Most obviously, this includes the people who shape public health and health care programs and policies. But it also includes the people who influence programs and policies in a wide variety of other aspects of the public sector (including education, housing, transportation, environment, and economic development), and in the non-profit and for-profit sectors (e.g., childcare, education, elder care). Successfully addressing these dissemination challenges will require a combination of methods including communication outreach, marketing, advocacy, illustrating with successful examples, technical assistance, and enabling the adaptation of evidence-based models to new circumstances.[180,181]

The Robert Wood Johnson Foundation's portfolio of Active Living initiatives provides an illustrative example.[182] Together, these programs seek to increase routine physical activity by disseminating evidence-based policy and environmental changes to American communities. Their methods include funding research studies to identify environmental factors and policies which influence physical activity; stimulating collaboration among professional associations to support elected and appointed government officials' efforts at promoting active living for their constituents; providing technical assistance to community partnerships to create and implement demonstration projects); and creating a "blueprint" that can be used by multiple organizations, associations and agencies to inform and support their change efforts. To highlight the need for change and the potential for success, each of the Active Living initiatives also conveys four key dissemination messages: (1) Physical activity has been engineered out of daily life; (2) As a result of inactivity, America is facing an obesity epidemic and related health problems; (3) By changing the places were we live and work, we can return physical activity to daily routines and reverse current health trends; (4) There is public support for creating activity-friendly places and this work is underway in some communities.[183]

### Maximizing the impact of existing communication and marketing resources

Although they may not think in these terms, most public health organizations currently make investments in – and have additional potential resources for – communication and marketing. Public health organizations should strive to enhance the impact of these investments because doing so can improve their agency's overall impact, even within current levels of funding.

With regard to communication, most public health organizations have actual resources in the form of communication expertise, information content, and some capacity to package and deliver that information to a variety of important audiences. Some public health organizations are well-positioned to use communication to target people-based fields of influence – based on their resources and capacity to reach people directly affected by health problems – while others are not. Conversely, based on their capacity to reach decision-makers at the local or distal level with credible information, some organizations are well positioned to use communication to target place-based fields of influence. Organizations should identify their current communication assets and determine how best to focus them.

Public health organizations should conduct a similar self-assessment with regard to marketing. An organization should identify: what products and services it currently offers people and other organizations; its capacity to improve those products and services – or to develop different products and services – based on feedback from current and potential customers; its capacity to deliver or distribute products and services to new priority populations; and finally, its capacity to promote its products or services to the people and organizations it wishes to serve.

Organizations are generally best served by developing programs that build on the strengths of their existing resources and core competencies, and by avoiding programs that require them to develop and sustain unrelated new resources. Once an organization is clear about the programs and information it wishes to deliver to its various customers, it can consider how its capacity can be extended through partnerships with other organizations. Partnering with other organizations – organizations that have a compelling reason to collaborate – is an important strategy both for enhancing impact, and for sustaining the initiative. Organizations can also build new resources and competencies, but doing so concurrent with developing new programs that requires those resources is challenging.

### The need for training

The public health workforce worldwide is currently under-trained in the critical functions of communication and marketing.[184] Schools of public health and other public health institutions must take seriously the need to identify necessary competencies in these disciplines, and to develop and deploy training approaches that meet the needs of both current and future public health professionals. While these training resources are likely to emerge sooner rather than later in nations of the developed world, we mustn't lose sight of the fact that similar training resources are even more desperately needed in nations of the developing world. International health organizations should rapidly develop and deploy a strategic plan to improve the communication and marketing competency of the public health workforce worldwide, especially in developing countries. The success or failure of public health initiatives often hinges on effective marketing and communication.

## Summary

• Communication and marketing are important tool kits for improving the public's health.

• These tool kits are uniquely well suited to advancing health in a manner consistent with an ecological model – as recommended by the Ottawa Charter – because each has the potential to influence people and places (i.e., environments).

• We suggest a practical framework by which to understand – and harness – the potential of communication and marketing to advance public health.

• Public health organizations should strive to enhance their competence in communication and marketing, because doing so can improve their impact even within current levels of funding.

• The public health workforce worldwide is currently under-trained in these critical competencies. International health organizations should rapidly develop and deploy a strategic plan to improve the communication and marketing competency of the public health workforce worldwide, especially in developing countries.

## Competing interests

The author(s) declare that they have no competing interests.

## Authors' contributions

Ed Maibach conceived and wrote the first draft of the manuscript. Lorien Abroms and Mark Marosits provided extensive input, feedback and editing on all sections of the paper.

## Pre-publication history

The pre-publication history for this paper can be accessed here:


